# Machine Learning Approaches to Radiogenomics of Breast Cancer using Low-Dose Perfusion Computed Tomography: Predicting Prognostic Biomarkers and Molecular Subtypes

**DOI:** 10.1038/s41598-019-54371-z

**Published:** 2019-11-28

**Authors:** Eun Kyung Park, Kwang-sig Lee, Bo Kyoung Seo, Kyu Ran Cho, Ok Hee Woo, Gil Soo Son, Hye Yoon Lee, Young Woo Chang

**Affiliations:** 1 Department of Radiology, Korea University Ansan Hospital, Korea University College of Medicine, 123 Jeokgeum-ro, Danwon-gu, Ansan-si, Gyeonggi-do 15355 Republic of Korea; 2 AI Center, Korea University Anam Hospital, Korea University College of Medicine, 73 Inchon-ro, Seongbuk-gu, Seoul 02841 Republic of Korea; 3 Department of Radiology, Korea University Anam Hospital, Korea University College of Medicine, 73, Goryeodae-ro, Seongbuk-gu, Seoul 02841 Republic of Korea; 4 Department of Radiology, Korea University Guro Hospital, Korea University College of Medicine, 148 Gurodong-ro, Guro-gu, Seoul 08308 Republic of Korea; 5 Division of Breast and Endocrine Surgery, Department of Surgery, Korea University Ansan Hospital, Korea University College of Medicine, 123 Jeokgeum-ro, Danwon-gu, Ansan-si, Gyeonggi-do 15355 Republic of Korea

**Keywords:** Cancer imaging, Computed tomography, Cancer imaging

## Abstract

Radiogenomics investigates the relationship between imaging phenotypes and genetic expression. Breast cancer is a heterogeneous disease that manifests complex genetic changes and various prognosis and treatment response. We investigate the value of machine learning approaches to radiogenomics using low-dose perfusion computed tomography (CT) to predict prognostic biomarkers and molecular subtypes of invasive breast cancer. This prospective study enrolled a total of 723 cases involving 241 patients with invasive breast cancer. The 18 CT parameters of cancers were analyzed using 5 machine learning models to predict lymph node status, tumor grade, tumor size, hormone receptors, HER2, Ki67, and the molecular subtypes. The random forest model was the best model in terms of accuracy and the area under the receiver-operating characteristic curve (AUC). On average, the random forest model had 13% higher accuracy and 0.17 higher AUC than the logistic regression. The most important CT parameters in the random forest model for prediction were peak enhancement intensity (Hounsfield units), time to peak (seconds), blood volume permeability (mL/100 g), and perfusion of tumor (mL/min per 100 mL). Machine learning approaches to radiogenomics using low-dose perfusion breast CT is a useful noninvasive tool for predicting prognostic biomarkers and molecular subtypes of invasive breast cancer.

## Introduction

Radiogenomics investigates the relationship between imaging phenotypes and underlying genes, their expression patterns, and mutations. The basic principle of radiogenomics is that imaging phenotypes are the result of processes occurring at the genetic and molecular levels^1^. Breast cancer is a heterogeneous disease that represents the accumulation of complex genetic alterations, and can have diverse prognosis and treatment responses. The goals of radiogenomics in breast cancer are to develop noninvasive imaging surrogate biomarkers and to predict the risks and outcomes of patient stratification for precise treatment. The radiogenomic approach to breast cancer has been recently developed mainly using dynamic contrast-enhanced magnetic resonance imaging (MRI) or mammography^2–7^. The associations of MRI or mammography with breast cancer gene sets, molecular subtypes, or recurrence scores have been investigated. A recent radiogenomic study using breast MRI and RNA sequencing by Yeh *et al*.^4^ demonstrated that tumor size characteristics are associated with proliferation and replication genetic pathways and better prognostic imaging features such as smaller size, increased sphericity, and that sharper margins are associated with immune pathway activation. Radiogenomic studies using mammography have shown the associations between breast parenchymal density with *UGT2B* gene variations and between radiographic texture analysis and *BRCA* gene mutations^6,7^.

The use of computed tomography (CT) to image the breast is limited because of the risk of high radiation exposure and poor image quality. However, CT has many advantages in oncology imaging. CT can be used to evaluate tumor angiogenesis using contrast agents and to assess lymph node or distant metastasis to lung, bone, or liver as well as breast itself. We recently developed a breast perfusion CT protocol using a low dose of 1.30–1.40 mSv, which was performed with the patient in the prone position, and reported the feasibility of quantifying tumor vascularity using this method^8^. Our preliminary results showed correlations between CT perfusion parameters and prognostic biomarkers and molecular subtypes, and between CT parameters and MRI enhancement characteristics. Low-dose breast perfusion CT requires a short scan time of only 3 minutes, produces satisfactory image quality, and can, therefore, be applied to patients who cannot undergo MRI because of implantable metallic devices, allergy to gadolinium, marked obesity, severe kyphoscoliosis, or claustrophobia.

We wanted to verify the application of low-dose perfusion CT to breast cancer radiogenomics by using a robust statistical method in a prospective study of breast cancer patients. Machine learning is a statistical tool that can allow computers to perform tasks by learning from examples without being explicitly programmed^9^. As data from electronic medical record become available, machine learning extracts knowledge from this data pool and produces output that can be used for individual outcome prediction analysis and clinical decision making. A few studies have applied a machine learning approach to radiogenomics and predictive analysis^10,11^. A machine learning approach allows for effective generalization of imaging data and patient stratification.

The purpose of our study was to investigate the clinical value of a machine learning approach to radiogenomics using low-dose perfusion breast CT for predicting prognostic biomarkers and molecular subtypes in invasive breast cancer. Although only 4 CT perfusion parameters with the maximum slope algorithm were extracted in the preliminary study^8^, the current study evaluated 18 quantitative CT parameters according to the maximum slope, deconvolution, and Patlak algorithms. The Patlak algorithm assumes the double-compartment method in which the intravascular and extravascular spaces are separate compartments^12,13^. The concentration of contrast agent within an organ at any time will have intravascular and extravascular components. This model quantifies the movement of the contrast agent from intravascular to extravascular space. The intravascular component is determined by the blood volume of the organ and the blood concentration of the contrast agent used at that time. The extravascular component is determined by the capillary permeability and the amount of contrast agent having passed through the organ. To create a CT feature-specific model, we used standard logistic regression and 5 machine learning models. We tried to identify the best machine learning model and which CT parameters are important for predicting prognostic biomarkers and molecular subtypes.

## Methods

### Ethics statement

This prospective study was approved by the Institutional Review Board of Korea University Ansan Hospital (2016AS0071 and 2017AS0037) and written informed consent was obtained from all patients. All methods were performed in accordance with the relevant guidelines and regulations.

### Patients

Perfusion CT was performed in 246 consecutive women who were scheduled to undergo treatment for invasive breast cancer from November 2016 to March 2019. All patients received a core needle biopsy and were not subjected to vacuum-assisted or excisional biopsies. Five of the 246 patients were excluded because of recent breast surgery within 2 years (n = 3), only ductal carcinoma *in situ* on final pathological examination after surgery (n = 1), and poor identification of a tumor in perfusion images because of small size (n = 1). Finally, a total of 241 breast cancer patients (age range, 25─84 years; mean, 51 years) were enrolled in this study.

### CT acquisition

Perfusion breast CT scans were performed according to the previous preliminary study^8^. One of 2 radiologists (B.K.S. and P.E.K.), who had 20 or 8 years’ experience in breast imaging, performed targeted ultrasound to localize the cancer before CT examination. After the cancer was identified on ultrasound, a dot skin marker (X-spot®; Beekley Medical, Bristol, CT, USA) was attached at the cancer site. If the patient had multiple cancers, the largest cancer was marked.

We used a spectral CT scanner (IQon Spectral CT; Philips Health Systems, Cleveland, OH, USA). For CT scanning in the prone position, an additional table pad with a rectangular hole to position the breast was placed on a standard CT table^8^. CT was performed at 80 kV tube voltage, 25 mAs or 30 mAs tube current, 64 × 0.625 mm collimation, 0.5 s rotation time, 512 × 512 matrix, and 5 mm slice thickness. 138 patients had a CT scan at 25 mAs and 103 patients at 30 mAs. The perfusion scan range was 40 mm along the z-axis, including skin markings on the cancers. Precontrast images were obtained to determine the scan range. After the perfusion range was determined, the skin markers were removed and 60 mL of contrast agent (Xenetix 350; Guerbet, Aulnay-sous-Bois, France) was administered at 4 mL/s. Scanning was performed 18 times at 3-second intervals followed by 4 times at 30-second intervals after the contrast administration. The CT dose index at 80 kVp and 25 mAs or 30 mAs using a 32 cm body phantom was 0.7 mGy or 0.9 mGy. The CT effective dose for each patient ranged from 1.01 to 1.40 mSv.

### CT analysis

**Figure 1:**
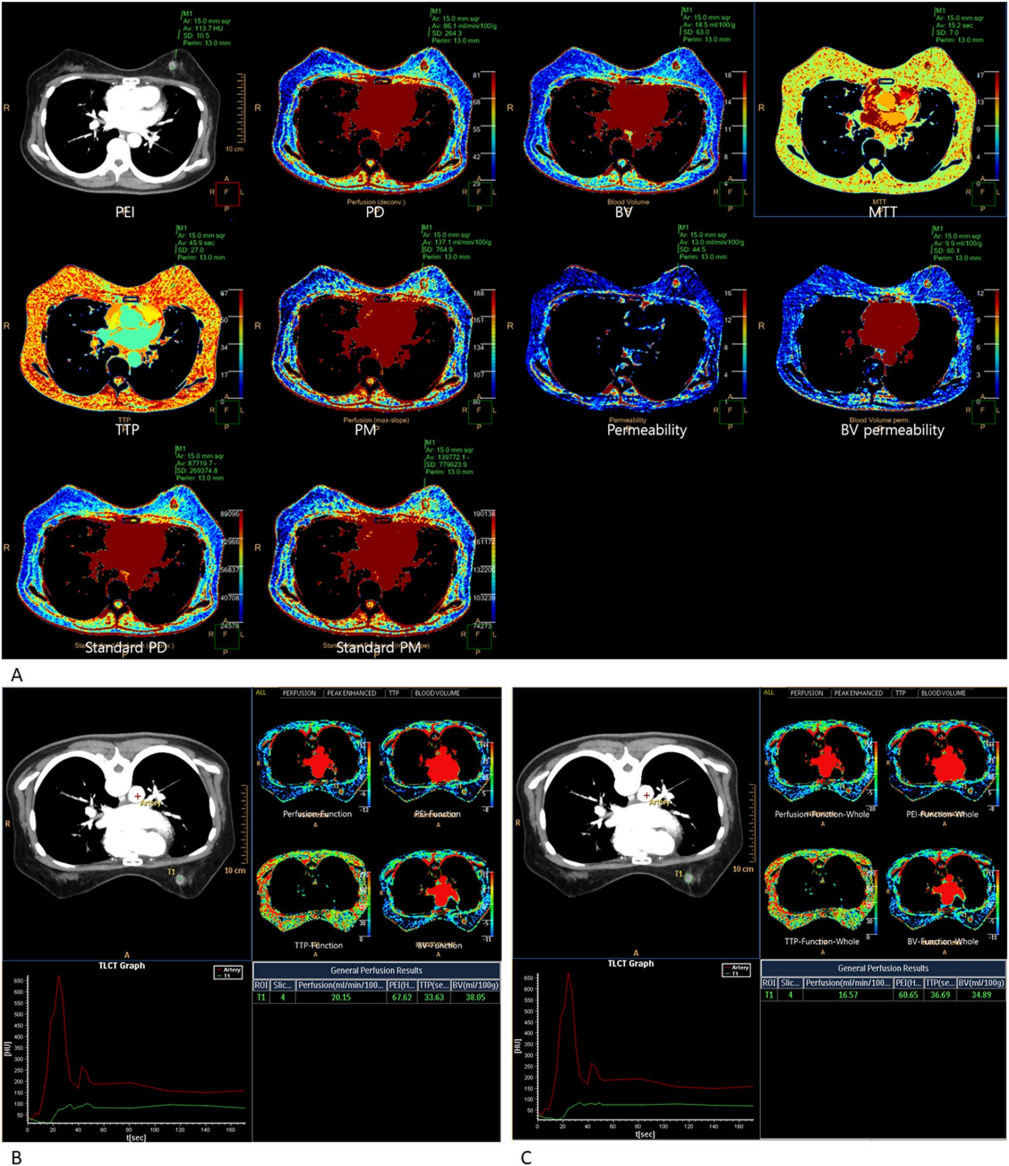
Evaluated 18 hemodynamic parameters on low-dose breast perfusion CT. (**A**) Ten parameters were obtained at the hot spot of the tumor using the Advanced Perfusion and Permeability Application software; peak enhancement intensity (PEI), perfusion on deconvolution model (PD), perfusion on maximum slope model (PM), blood volume (BV), mean transit time (MTT), time to peak (TTP), permeability (Permeability), blood volume permeability (BV permeability), standardization of perfusion on deconvolution model (Standard PD), and standardization of perfusion on maximum slope model (Standard PM). (**B**) Four parameters were obtained at the hot spot of the tumor using the Functional CT software; perfusion (Perfusion-Function), PEI (PEI-Function), TTP (TTP-Function), and BV (BV-Function). (**C**) Four parameters were obtained at the whole tumor range using the Functional CT software; perfusion of whole breast cancer (Perfusion-Function-Whole), PEI of whole breast cancer (PEI-Function-Whole), TTP of whole breast cancer (TTP-Function-Whole), and BV of whole breast cancer (BV-Function-Whole).

The CT data were sent to a dedicated workstation (Extended Brilliance Workspace; Philips Health Systems). All 18 perfusion parameters (independent variables) were obtained using the 2 software programs (Table 1). The time-attenuation curves and perfusion color maps of the cancer were computed automatically using a commercial software (Functional CT (FUNCTION); Philips Health Systems) and a prototype (Advanced Perfusion and Permeability Application (APPA); Philips Health Systems). (Fig. 1).

**Table 1:** List of evaluated perfusion CT parameters.

CT perfusion parameters	Features
PEI (Hounsfield Units)^*^	Peak enhancement intensity by APPA: The peak enhancement due to injected contrast agent
PD (mL/min per 100 mL)^*^	Perfusion on deconvolution model by APPA: Blood flow through the vasculature in a defined tissue or mass volume on deconvolution model
PM (mL/min per100 mL)^*^	Perfusion on maximum slope model by APPA: Blood flow through the vasculature in a defined tissue or mass volume on maximum slope model
BV (mL/100 g)^*^	Blood volume by APPA: The total blood volume over the region during the period of the scan and it is determined by the area under the time-attenuation curve
MTT (seconds)^*^	Mean transit time by APPA: Average transit time of contrast agent in a given tissue
TTP (seconds)^*^	Time to peak by APPA: The time it takes for the peak enhancement to be reached
Permeability (mL/min per 100 g)^*^	Permeability by APPA: The flow of molecules through the capillary membranes in a certain volume of tissue
BV permeability (mL/100 g)^*^	Blood volume permeability by APPA: The blood volume passed through the contrast agent from the intravascular space into the extravascular space
Standard PD (mL/min per 100 mL)^*^	Standardization of perfusion on deconvolution model by APPA: Calculated perfusion value based on four inputs including cardiac output, body weight of the patient, volume and density of the injected contrast agent, and conversion factor (contrast agent density unit to HU [HU/(mg/mL)] on deconvolution model
Standard PM (mL/min per 100 mL)^*^	Standardization of perfusion on maximum slope model by APPA: Calculated perfusion value based on four inputs including cardiac output, body weight of the patient, volume and density of the injected contrast agent, and conversion factor (contrast agent density unit to HU [HU/(mg/mL)] on maximum slope model
Perfusion-Function (mL/min per 100 mL)^*^	Perfusion by FUNCTION
PEI-Function (Hounsfield Units)^*^	Peak enhancement intensity by FUNCTION
TTP-Function (seconds)^*^	Time to peak by FUNCTION
BV-Function (mL/100 g)^*^	Blood volume by FUNCTION
Perfusion-Function-Whole (mL/min per 100 mL)	Perfusion of whole tumor by FUNCTION
PEI-Function-Whole (Hounsfield Units)	Peak enhancement intensity of whole tumor by FUNCTION
TTP-Function-Whole (seconds)	Time to peak of whole tumor by FUNCTION
BV-Function-Whole (mL/100 g)	Blood volume of whole tumor by FUNCTION

^*^Perfusion parameters measured by ROI placed for breast cancer covering the hot spot of the tumor.

*CT* computed tomography, *APPA* advanced perfusion and permeabiliy application software, *FUNCTION* Functional CT software.

Ten parameters were obtained using the APPA program (Fig. 1A); peak enhancement intensity (PEI; Hounsfield units, HU), perfusion on deconvolution model (PD; mL/min per 100 mL), perfusion on maximum slope model (PM; mL/min per 100 mL), blood volume (BV; mL/100 g), mean transit time (MTT; seconds), time to peak (TTP; seconds), permeability (Permeability; mL/min per 100 g), blood volume permeability (BV permeability; mL/100 g), standardization of perfusion on deconvolution model (Standard PD; mL/min per 100 mL), and standardization of perfusion on maximum slope model (Standard PM; mL/min per 100 mL). Standardization meant calculated perfusion values based on cardiac output, body weight, volume and density of the contrast agent, and conversion factors (contrast agent density unit to HU).

Eight parameters were obtained using FUNCTION program. 4 parameters were measured at the tumor hot spots (Fig. 1B), and 4 were measured over the whole tumor (Fig. 1C); perfusion (Perfusion-Function; mL/min per 100 mL), PEI (PEI-Function; HU), TTP (TTP-Function; seconds), BV (BV-Function; mL/100 g), perfusion of whole breast cancer (Perfusion-Function-Whole; mL/min per 100 mL), PEI of whole breast cancer (PEI-Function-Whole; HU), TTP of whole breast cancer (TTP-Function-Whole; seconds), and BV of whole breast cancer (BV-Function-Whole; mL/100 g).

A perfusion map of breast cancer was obtained by (1) manually selecting the images between the beginning and end of enhancement in the descending aorta area, (2) obtaining the reference artery input curve by placing a region of interest (ROI) in the aorta, and (3) placing a ROI in the tumor hot spot or the whole tumor range. Perfusion parameters for each patient were measured 3 times at intervals of 3 months by the radiologist (E.K.P.). The tumor size was measured on CT scans using the maximum diameter of the enhancing tumor, and the size was then classified into 2 groups for statistical analysis: ≤20 mm or >20 mm. The tumor size ranged from 6.0 mm to 82.0 mm (mean, 23.8 ± 13.4 mm).

### Histopathological evaluation

We reviewed the histopathological reports for evaluation of prognostic biomarkers and molecular subtypes (dependent variables) of breast cancer. Lymph node status, tumor grade, estrogen receptor (ER), progesterone receptor (PR), human epidermal growth factor receptor 2 (HER2), and Ki67 were dichotomized for statistical analysis. Lymph node status was divided into positive or negative metastasis. Tumor grade was dichotomized as low (grade 1 and 2) and high (grade 3)^14,15^. The immunohistochemical results of ER, PR, HER2, and Ki67 were classified as positive or negative. The Allred scoring system was used for ER and PR status, and a score of >2 was considered positive. Positive HER2 overexpression was considered in 3+ immunohistochemical staining or 2+ immunohistochemical staining and HER2 gene amplification in silver-stained *in situ* hybridization. The Ki67 index was considered positive when the expression was >20%.

The molecular subtypes of breast cancer divided into 4 categories: luminal A (ER or PR+, HER2−, and Ki67−); luminal B (ER or PR+, HER2−, and Ki67+, or ER or PR+, HER2+, and Ki67+); HER2 overexpression (ER−, PR−, and HER2+); or triple negative cancer (ER−, PR−, and HER2−).

### Statistical analysis

The primary goal of this study was to evaluate whether popular machine learning models, that is, decision tree, naïve Bayes, random forest, support vector machine (SVM) and artificial neural network (ANN) based on CT perfusion features, can predict prognostic biomarkers and molecular subtypes of breast cancer. A decision tree comprised (a) internal nodes (each denoting a test of an attribute or independent variable), (b) branches (each denoting an outcome of the test), and (c) terminal nodes (each representing a class label or dependent variable). A naïve Bayesian classifier is a predictor based on Bayes’ theorem. A random forest creates many training sets, trains many decision trees, and makes a prediction with a majority vote (bootstrap aggregation). An SVM makes a prediction by maximizing a margin among hyperplanes separating data. The ANN of this study included 1 input layer, 2 hidden layers, and 1 output layer with 9774 neurons as data units in the input layer, 15 in each hidden layer, and 4 (or 2) in the output layer, i.e., 4 for the molecular subtypes and 2 for the dichotomized status of a prognostic biomarker for breast cancer. Here, the number of neurons in the input layer, 9774, was derived from the multiplication of 18 and 543, the numbers of attributes and cases in the training set, respectively. Neurons in the input or previous hidden layer were combined with weights in the next hidden or output layer (feedforward algorithm). The weights in the output layer and its previous hidden layers were then adjusted according to how much they contributed to the loss of the ANN, i.e., a gap between the actual and predicted class labels (backpropagation algorithm). Initially, the weights were set as small random numbers around 0, and the feedforward and backpropagation algorithms were iterated until certain criteria were met for the accurate prediction of a class label^16^.

The model input variables included 18 quantitative CT parameters (Table 1). The model output variables were prognostic biomarkers and molecular subtypes of breast cancer. The data on 723 cases were divided into training and test sets at a 75:25 ratio. The models were built based on the training set of 543 cases, and the trained models were then validated based on the test set of 180 cases. Two often used criteria were introduced for validating the trained models and finding the best prediction model; accuracy, which represents the ratio of correct predictions among 180 cases in the test set, and the area under the receiver-operating characteristic curve (AUC), which represents the plot of the true positive rate (or sensitivity) vs. the false positive rate (or 1 – specificity). A single 75:25 split of the training and test sets would reduce the validity and generalizability of the results. For this reason, the random split and the statistical analysis were repeated 50 times and their average accuracy and AUC were calculated for each of six statistical methods, i.e., logistic regression and 5 machine learning models above^17^. Here, an additional step with a validation set is considered to be essential when hyper-parameter tuning is essential as in the case of deep learning models with many layers. In this case, (1) the validation set is held back from the training set besides the test set; (2) the validation set is used for tuning hyper-parameters of a certain model (e.g., the learning rate in a deep learning model); and (3) the test set is used for selecting the best model based on performance measures such as accuracy and the AUC. However, this additional step with the validation set can be skipped when hyper-parameter tuning is not essential, as in this study. Therefore, a validation set was not used here. Finally, the importance ranking of CT parameters for predicting the prognostic biomarkers and the molecular subtypes were derived from the variable importance measure of the best prediction model, which gave an accuracy (or mean impurity) gap between the complete model and a model excluding a certain variable. Python 3.52 was used for the analysis in April 2019.

## Results

### Descriptive statistics of prognostic biomarkers, molecular subtypes, and CT perfusion parameters

Table 2 shows the descriptive statistics for the prognostic biomarkers and molecular subtypes of invasive breast cancer. The statistics for the positive prognostic biomarkers were 43%, 67%, 63%, 21%, and 53% for lymph node, ER, PR, HER2, and Ki67, respectively. Similarly, the percentages of cases with molecular subtypes, luminal A, luminal B, HER2 overexpression, and triple negative cancers were 41%, 29%, 17%, and 13%, respectively. Table 3 indicates the descriptive statistics for the 18 CT perfusion parameters expressed as average, minimum, maximum, and 25%, 50%, and 75% values.

**Table 2:** Descriptive statistics for prognostic biomarkers and molecular subtypes.

Dependent variable	Count	Percentage (%)
Lymph node		
Negative	414	57
Positive	309	43
Tumor grade		
Low	459	63
High	264	37
Tumor size		
≤20	360	50
>20	363	50
ER		
Negative	237	33
Positive	486	67
PR		
Negative	270	37
Positive	453	63
HER2		
Negative	570	79
Positive	153	21
Ki67		
Negative	339	47
Positive	384	53
Molecular subtype		
Luminal A	294	41
Luminal B	207	29
HER2 overexpression	126	17
Triple negative	96	13

*ER* estrogen receptor, *PR* progesterone receptor, *HER2* human epidermal growth factor receptor 2.

**Table 3:** Descriptive statistics for CT perfusion parameters.

Independent variable^*^	Mean	SD	Min	25%	50%	75%	Max
PEI (Hounsfield Units)	112.15	34.03	20.00	94.10	112.60	130.80	251.60
PD (mL/min per 100 mL)	108.14	36.11	31.20	81.85	102.00	128.60	356.80
PM (mL/min per100 mL)	219.05	89.99	79.30	159.15	200.90	257.40	950.10
BV (mL/100 g)	20.57	6.81	4.80	15.90	19.80	23.75	51.30
MTT (seconds)	14.90	5.97	7.80	12.60	14.70	16.60	156.20
TTP (seconds)	48.10	8.42	26.40	41.60	47.60	54.35	67.10
Permeability (mL/min per 100 g)	14.80	8.83	0.10	10.50	13.80	17.30	118.40
BV permeability (mL/100 g)	13.29	17.44	0.00	5.40	10.50	17.25	371.50
Standard PD (mL/min per 100 mL)	10.19	3.21	3.12	7.80	9.90	12.08	24.54
Standard PM (mL/min per 100 mL)	20.67	8.00	6.51	14.68	19.55	24.85	63.61
Perfusion-Function (mL/min per 100 mL)^*^	31.81	31.30	2.22	9.23	22.15	45.36	255.45
PEI-Function (Hounsfield Units)	71.87	28.08	12.08	53.74	68.91	84.58	192.24
TTP-Function (seconds)	55.97	49.32	3.05	18.34	30.56	92.75	366.69
BV-Function (mL/100 g)	42.83	36.64	0.58	24.28	33.39	48.62	268.54
Perfusion-Function-Whole (mL/min per 100 mL)	12.97	17.01	0.76	3.26	5.59	15.15	134.82
PEI-Function-Whole (Hounsfield Units)	44.40	19.46	4.25	31.67	42.82	53.60	193.63
TTP-Function-Whole (seconds)	89.29	54.22	6.11	27.51	95.81	135.56	168.19
BV-Function-Whole (mL/100 g)	28.75	26.71	0.16	15.63	22.81	32.59	256.66

^*^The meanings of independent variables are described in Table 1.

*CT* computed tomography, *SD* standard deviation, *Min* minimum value, *25%* 25 percentile value of the distribution, *50%* 50 percentile value of the distribution, *75%* 75 percentile value of the distribution, *Max* maximum value.

### Diagnostic performance of the logistic regression and machine learning models for predicting prognostic biomarkers and molecular subtypes

Table 4 shows the diagnostic performance of the logistic regression and 5 machine learning models in terms of accuracy and AUC values, averaged over the 50 random splits and statistical analyses described above. Indeed, Fig. S1 shows the AUCs from one of the 50 analyses. In terms of accuracy and the AUC, the random forest model was the best model for predicting prognostic biomarkers and molecular subtypes of breast cancer. The accuracy was higher for the random forest model than for the logistic regression model by 13% on average: 78% vs. 62% for lymph node status, 80% vs. 67% for tumor grade, 77% vs. 64% for tumor size, 82% vs. 70% for ER status, 78% vs. 66% for PR status, 83% vs. 78% for HER2 status, 77% vs. 65% for Ki67 status, and 66% vs. 48% for molecular subtypes. The random forest model was a better performer than the logistic regression model, as shown by the average 0.17 difference in AUC: 0.86 vs. 0.66 for lymph node status, 0.88 vs. 0.71 for tumor grade, 0.85 vs. 0.69 for tumor size, 0.88 vs. 0.73 for ER status, 0.85 vs. 0.68 for PR status, 0.88 vs. 0.69 for HER2 status, 0.85 vs. 0.70 for Ki67 status, and 0.82 vs. 0.69 for molecular subtypes.

**Table 4:** Average performance of logistic regression and machine learning models for predicting prognostic biomarkers and molecular subtypes.

	Lymph node		Tumor grade		Tumor size		ER		PR		HER2		Ki67		Molecular subtype	
	Accuracy	AUC	Accuracy	AUC	Accuracy	AUC	Accuracy	AUC	Accuracy	AUC	Accuracy	AUC	Accuracy	AUC	Accuracy	AUC
Logistic regression	62%	0.66	67%	0.71	64%	0.69	70%	0.73	66%	0.68	78%	0.69	65%	0.70	48%	0.69
Decision tree	66%	0.65	71%	0.69	65%	0.65	72%	0.69	69%	0.67	77%	0.67	66%	0.66	50%	0.63
Naïve Bayes	53%	0.58	63%	0.70	58%	0.63	68%	0.71	65%	0.65	72%	0.69	59%	0.69	49%	0.70
Random forest	**78%**	**0.86**	**80%**	**0.88**	**77%**	**0.85**	**82%**	**0.88**	**78%**	**0.85**	**83%**	**0.88**	**77%**	**0.85**	**66%**	**0.82**
SVM	57%	0.34	63%	0.35	48%	0.42	67%	0.44	64%	0.39	79%	0.47	53%	0.35	41%	0.65
ANN	64%	0.68	68%	0.73	65%	0.71	75%	0.77	69%	0.72	76%	0.73	66%	0.71	35%	0.72

*ER* estrogen receptor, *PR* progesterone receptor, *HER2* human epidermal growth factor receptor 2, *AUC* the area under the receiver-operating-characteristic curve, *SVM* support vector machine, *ANN* artificial neural network.

### Variable importance of CT parameters in predicting prognostic biomarkers and molecular subtypes

According to the importance of CT variables from the 50 random forest model, PEI, TTP, BV permeability, Perfusion-Function, and Perfusion-Function-Whole were the most important parameters for predicting prognostic biomarkers and molecular subtypes of breast cancer (Table 5). These results were based on the number of times a certain CT parameter ranked within the top 5 for the predictions (top 5 criteria). These CT parameters were also the most important parameters based on the number of times a certain CT parameter ranked within the top 10 for the predictions (top 10 criteria). PEI-Function, PM, Permeability, TTP-Function-Whole, and BV-Function-Whole satisfied the top 10 criteria.

**Table 5:** Variable importance of CT parameters from the random forest in predicting prognostic biomarkers and molecular subtypes.

Independent variable^*^	Lymph node	Tumor grade	Tumor size	ER	PR	HER2	Ki67	Molecular subtype
PEI (Hounsfield Units)	0.0589^†^	0.0685^†^	0.0860^†^	0.0498	0.0520	0.0681^†^	0.0664^†^	0.0614^†^
PD (mL/min per 100 mL)	0.0564	0.0505	0.0508	0.0433	0.0430	0.0408	0.0552	0.0478
PM (mL/min per100 mL)	0.0583^†^	0.0444	0.0507	0.0445	0.0497	0.0460	0.0433	0.0484
BV (mL/100 g)	0.0433	0.0479	0.0455	0.0391	0.0448	0.0436	0.0478	0.0479
MTT (seconds)	0.0413	0.0458	0.0487	0.0413	0.0488	0.0480	0.0399	0.0430
TTP (seconds)	0.0420	0.0771^†^	0.0566	0.0689	0.0743^†^	0.0743^†^	0.0686^†^	0.0665^†^
Permeability (mL/min per 100 g)	0.0491	0.0462	0.0482	0.0731^†^	0.0835^†^	0.0526	0.0505	0.0651^†^
BV permeability (mL/100 g)	0.0428	0.0668^†^	0.0462	0.0830^†^	0.0675^†^	0.0718^†^	0.1054^†^	0.0823^†^
Standard PD (mL/min per 100 mL)	0.0530	0.0547	0.0450	0.0463	0.0483	0.0460	0.0457	0.0454
Standard PM (mL/min per 100 mL)	0.0474	0.0483	0.0459	0.0413	0.0532	0.0439	0.0406	0.0464
Perfusion-Function (mL/min per 100 mL)	0.0500	0.0576	0.0752^†^	0.0727^†^	0.0666^†^	0.0493	0.0625^†^	0.0657^†^
PEI-Function (Hounsfield Units)	0.0607^†^	0.0612	0.0556	0.0550	0.0485	0.0619	0.0631^†^	0.0575
TTP-Function (seconds)	0.0435	0.0377	0.0491	0.0642	0.0653	0.0460	0.0506	0.0555
BV-Function (mL/100 g)	0.0556	0.0537	0.0745^†^	0.0391	0.0393	0.0418	0.0598	0.0526
Perfusion-Function-Whole (mL/min per 100 mL)	0.0757^†^	0.0728^†^	0.0586^†^	0.0686^†^	0.0657^†^	0.0848^†^	0.0487	0.0574
PEI-Function-Whole (Hounsfield Units)	0.0638^†^	0.0451	0.0652^†^	0.0436	0.0519	0.0721^†^	0.0581	0.0471
TTP-Function-Whole (seconds)	0.0556	0.0645^†^	0.0457	0.0808^†^	0.0561	0.0564	0.0381	0.0550
BV-Function-Whole (mL/100 g)	0.0469	0.0571	0.0525	0.0455	0.0416	0.0527	0.0556	0.0550

^*^The meanings of independent variables are described in Table 1.

^†^Top 5 important variables with the highest variable importance scores in predicting a prognostic biomarker or molecular subtype.

*CT* computed tomography, *ER* estrogen receptor, *PR* progesterone receptor, *HER2* human epidermal growth factor receptor 2.

The importance rankings of PEI were 4^th^, 3^rd^, 1^st^, 5^th^, 3^rd^, and 5^th^ for lymph node status, tumor grade, tumor size, HER2 status, Ki67 status, and molecular subtypes, respectively. TTP ranked 1^st^, 2^nd^, 2^nd^, 2^nd^, and 2^nd^ among the most important predictors for tumor grade, PR status, HER2 status, Ki67 status, and molecular subtypes, respectively. BV permeability ranked 4^th^, 1^st^, 3^rd^, 4^th^, 1^st^, and 1^st^ among the most important predictors for tumor grade, ER status, PR status, HER2 status, Ki67 status, and molecular subtypes, respectively. The measures of Perfusion-Function-Whole were 1^st^, 2^nd^, 5^th^, 5^th^, 5^th^, and 1^st^ for lymph node, tumor grade, tumor size, ER, PR, and HER2, respectively. The measures for Perfusion-Function were 2^nd^, 4^th^, 4^th^, 5^th^, and 3^rd^ for predicting tumor size, ER status, PR status, Ki67 status, and molecular subtypes, respectively.

The multinomial logistic regression results yielded useful information about the direction and magnitude of the effects of the top 5 CT parameters on the prognostic biomarkers and molecular subtypes (Table S1). For example, a 10-second increase in TTP would increase the odds of ER being positive (vs. negative) by 78%. A 10-second decrease in TTP would lead to a 137% increase in the odds of HER2 overexpression cancer (vs. luminal A type cancer). Similarly, a 10-second decrease in TTP would result in a 97% increase in the odds of triple negative cancer (vs. luminal A type cancer). An increase of 10 mL/min per 100 mL in Perfusion-Function would increase the odds of HER2 overexpression cancer (vs. luminal A type cancer) by 25%. Indeed, the results of univariate analysis were consistent with their multivariate counterparts in general (Supplementary Table S2). A difference in the mean of a CT parameter was positive (or negative) in cases when the odds ratio was greater (or smaller) than 1 with respect to a prognostic biomarker/molecular subtype. For instance, a 10-second decrease in the TTP would result in a 97% increase in the odds of triple negative cancer (vs. luminal type A cancer). Likewise, the TTP mean was significantly smaller for triple negative cancer (44.45) than for luminal type A cancer (51.14). These results would supplement the random forest, the best model for predicting the prognostic biomarkers and the molecular subtypes in terms of accuracy and the AUC.

## Discussion

Until recently, radiogenomic investigations of breast cancer have been carried out using MRI or mammography. This prospective study has shown the possibility of using CT for radiogenomic investigations of different breast cancer subtypes. Our CT protocol provides a quantitative variety of perfusion parameters associated with tumor angiogenesis, with fairly low radiation doses while maintaining high image quality. In addition, the feasibility of this innovative and convenient CT technology was evaluated using a powerful statistical method, a machine learning approach. Our results show that quantitative perfusion CT parameters are associated with the prognostic biomarkers and molecular subtypes of breast cancer, and that the random forest model was the best machine learning model in terms of accuracy and AUC for predicting the prognosis using CT parameters. The random forest model had better accuracy and AUC than the logistic regression model. The accuracy of the random forest model was on average 13% higher than that of the logistic regression model, and the mean AUC difference between the random forest and logistic regression models was 0.17. In addition, we identified the 5 most important CT variables for prediction: PEI, TTP, BV permeability, Perfusion-Function, and Perfusion-Function-Whole.

Our study shows 3 key advances in radiogenomics of breast cancer. First, we have provided an innovative breast CT protocol that measures tumor vascularity using a very low radiation dose. In our previous preliminary study of low-dose perfusion CT, we used 80 kVp tube voltage and 30 mA tube current, and the CT effective dose of the protocol ranged from 1.30 to 1.40 mSv^8^. We have continued to strive to reduce the radiation exposure while maintaining the quality of the images, and we performed CT scans with tube currents down to 25 mAs. Of a total of 241 patients, 138 underwent the CT scan at 25 mAs and the remaining 103 underwent the CT scan at 30 mAs. The CT protocol using a 25mAs tube current reduced the effective radiation dose to 1.01 mSv. In the United States, the average annual effective dose of natural background radiation is about 3 mSv^18^. The average effective dose of 2-view film-screen mammography is 0.56 mSv and that of digital mammography is 0.44 mSv^19^. Therefore, this low-dose perfusion CT protocol is clinically applicable in terms of radiation exposure. If a flexible scan range customized for breast cancer size is available, the effective dose should be reduced to less than 1 mSv for small breast cancers. Reducing the frequency of the acquisition to create time-attenuation curves and developing radiation-reducing algorithms and reconstruction programs may reduce the radiation dose even further.

Second, we have demonstrated the clinical value of quantitative hemodynamic parameters in perfusion CT for predicting biomarkers and molecular subtypes of breast cancer using machine learning models. We evaluated the performance of 5 machine learning models and standard logistic regression for prediction using the measurement of accuracy and AUC values. The best model in this study was the random forest model. The accuracy and AUC values were consistently higher for the random forest model than the standard logistic regression model. In particular, for predicting the molecular subtypes, luminal A, luminal B, HER2 overexpression, or triple negative cancer, the random forest model had superb results, as shown by the comparisons of the random forest vs. logistic regression models: 66% vs. 48% for accuracy and 0.82 vs. 0.69 for AUC.

The random forest model is known for its robust performance and strong generalization power from bagging (or bootstrap aggregation)^20^. As noted in the Statistical Analysis section above, a random forest model creates many training sets, trains many decision trees, and makes a prediction with a majority vote (bagging). The random forest of this study comprised 1000 decision trees. Intuitively, a majority vote made by 1000 doctors would be more reliable than a vote made by 1 doctor. In a similar vein, a majority vote made by 1000 decision trees would be more reliable than a vote made by a single machine learning method. To our knowledge, no research has investigated the value of machine learning approaches to radiogenomics using breast CT scanning, breast perfusion imaging, or breast imaging parameters reflecting tumor vascularity to predict biomarkers and molecular subtypes of breast cancer. The results of this study confirm the robust performance and strong generalization power of the random forest model using bagging for the clinical purpose of disease diagnosis and prognosis.

Third, our machine learning approach identified important CT hemodynamic parameters in relation to the treatment and prognostic indicators of breast cancer. In the previous preliminary study of low-dose perfusion CT^8^, 4 CT parameters were obtained with 1 perfusion algorithm (maximum slope) of the FUNCTION analysis software. In the current study, we extracted 18 CT parameters with 3 perfusion algorithms (maximum slope, deconvolution, and Patlak). We sought to identify which CT parameters are more important for predicting biomarkers and molecular subtypes using a machine learning approach. According to the importance of CT variables in the random forest model, PEI, TTP, BV permeability, Perfusion-Function, and Perfusion-Function-Whole were the 5 most important CT parameters for prediction overall. The following CT parameters were valuable for predicting particular prognostic biomarkers or the molecular subtypes: BV permeability, TTP-Function-Whole, Permeability, Perfusion-Function, and Perfusion-Function-Whole for ER status; Perfusion-Function-Whole, TTP, PEI-Function-Whole, BV permeability, and PEI for HER2 status; BV permeability, TTP, PEI, PEI-Function, and Perfusion-Function for Ki67 status; and BV permeability, TTP, Perfusion-Function, Permeability, and PEI for the molecular subtypes.

Tumor angiogenesis plays a vital role in the growth and metastasis of tumors^21^. In breast cancer, angiogenesis is associated with tumor hypoxia and is an independent predictor of overall disease-free survival^22,23^. New tumor vessels in malignant tumors generally contain immature microvessels^24,25^, which increase permeability, and arteriovenous shunts and hyperpermeable vessels, which increase the blood flow within the tumor. Therefore, aggressive tumors can demonstrate a faster time to reach a peak enhancement. Our multinomial logistic regression results showed that a decrease in TTP would lead to a rise in the odds of breast cancer with positive lymph node metastasis, higher tumor grade, larger tumor size, hormone receptor negativity, HER2 positivity, or higher Ki67 level. A decrease in TTP would result in a rise in the odds of HER2 overexpressing cancer compared to luminal type cancer. Our results also demonstrate the importance of TTP as a noninvasive image parameter for predicting prognostic biomarkers and molecular subtypes of breast cancer. TTP ranked within the top 5 for the predictions of tumor grade, PR status, HER2 status, Ki67 status, and the molecular subtypes.

Blood flow through the vasculature is also related to the velocity within the tumor. In this study, CT parameters that indicated blood flow were PD, PM, Standard PD, Standard PM, Perfusion-Function, and Perfusion-Function-Whole. These are obtained from various perfusion algorithms (maximum slope or deconvolution), various analyzing software (FUNCTION or APPA), various tumor regions (hot spots or whole tumor), and the use of standardization based on cardiac output, body weight, volume and density of the contrast agent, and conversion factors. Perfusion-Function and Perfusion-Function-Whole ranked within the top 5 in predicting biomarkers and molecular subtypes of breast cancer. The multinomial logistic regression results demonstrated that a growth in Perfusion-Function (i.e., blood flow at the hot spot of tumor) would lead to a rise in the odds of cancers with the worse prognostic factors such as positive lymph node metastasis, larger tumor size, hormone receptor negativity, HER2 positivity, or higher Ki67 level, and that a growth in Perfusion-Function would result in a rise in the odds of HER2 overexpressing cancer or triple negative cancer compared to luminal type cancer. These results were consistent with the previous preliminary study^8^.

Immature microvessels in tumors are fragile and hyperpermeable. Vascular permeability indicates the capacity of the blood vessel walls to allow for the flow of molecules or cells in and out of the vessel. In cancer, the vascular wall is important because it acts a barrier to drugs, nutrients, and immune cells. Disorganized and leaky vessel networks cause tumor cell extravasation, blood flow disturbance, and inflammatory cell infiltration, and vascular permeability is associated with tumor progression and can affect drug delivery. In perfusion CT, the permeability is evaluated using the Patlak algorithm^13^. In this study, after administration of the contrast agent, CT was performed 18 times at 3-second intervals followed by 4 times at 30-second intervals. Four scans were taken at 84, 114, 144, and 174 seconds to obtain information about permeability. We measured 2 parameters related to permeability using the Patlak algorithm; Permeability and BV permeability. Permeability indicates the flow of molecules through the capillary membranes in a certain volume of tissue (mL/min per 100 g). BV permeability indicates BV and the movement of the contrast agent from the intravascular space into the extravascular space (mL/100 g). The Patlak algorithm assumes the double-compartment method in which the intravascular and extravascular spaces are separate compartments. This algorithm quantifies the movement of the contrast agent from the intravascular space into the extravascular space and is described in the following equation:

$${\rm{y}}={\rm{b}}+{\rm{a}}\cdot {\rm{x}},$$

$$\frac{C(t)}{Cb(t)}=rbv+Pm\frac{{\int }_{(0)}^{(t)}Cb(t)\cdot dt}{Cb(t)},$$

where b is the blood volume, a is the permeability, C(t) is the tissue time-attenuation curve, Cb(t) is the input artery time-attenuation curve, rbv is the tissue BV, and Pm is the permeability coefficient. Two required parameters, y and x, are calculated using linear regression, which finds the line that best fits the measured data. In the algorithm application, this calculation is performed for each voxel in the scanned volume. Based on our results, BV permeability was a more important CT parameter for predicting biomarkers and molecular subtypes than permeability. In addition, the logistic regression results showed that an increase in BV permeability values would lead to a rise in the odds of cancers with the worse prognostic factors such as higher tumor grade, hormone receptor negativity, or higher Ki67. However, the relationship between permeability and prognostic biomarkers was not consistent in logistic regression analysis and further studies are needed to determine the clinical relevance of the two permeability-related parameters.

This study had several limitations. First, this study was performed using one spectral CT device from one institution. However, the preliminary study of low-dose breast perfusion CT has already shown the feasibility to quantify tumor vascularity and significant correlations of CT parameters with prognostic biomarkers and molecular subtypes in 70 patients with invasive breast cancers^8^. In this prospective study, we measured CT parameters three times at 3-month intervals for each patient and generated a large data set with 723 cases from 241 invasive breast cancer patients. Second, we evaluated fewer imaging parameters than did the general studies related to radiomics or radiogenomics because we only extracted perfusion-related imaging phenotypes for the assessment of tumor angiogenesis. We extracted 18 imaging features given that perfusion CT scans can only measure perfusion-related variables. However, we calculated perfusion CT parameters using all existing applicable perfusion CT algorithms in this study, the maximum slope, deconvolution, and Patlak. Therefore, our study has obtained the most perfusion parameters in perfusion CT studies including breast as well as other organs. Moreover, this study introduced innovative machine learning methods to demonstrate that low-dose perfusion breast CT is a useful noninvasive tool for predicting prognostic biomarkers and molecular subtypes of invasive breast cancer. Third, we used hand-drawn ROIs in the evaluation of perfusion parameters in the tumor hotspots. Future development of automatic selection and calculation software are helpful for overcoming subjectivity. Fourth, this study was designed to make predictions based on a predetermined set of predictors, namely 18 CT parameters. Conducting various experiments based on random sets of predictors and comparing their performance measures would be a good topic for further research.

## Conclusion

This study demonstrates that a machine learning approach to radiogenomics using low-dose perfusion CT is a useful noninvasive tool for predicting prognostic biomarkers and molecular subtypes of invasive breast cancer. The machine learning random forest model was the best model for predictions with an average accuracy improvement of 13% and an average AUC improvement of 0.17 compared to logistic regression. The combination of innovative and convenient CT technology and a robust statistical model is a promising tool for radiogenomics of breast cancer and can help with risk stratification and precise treatment.

## Supplementary information

Supplementary tables and figure

## Data Availability

The datasets generated and/or analyzed during the current study are available from the corresponding author on reasonable request.
