# Supplementary material for: Machine Learning Approaches to Radiogenomics of Breast Cancer using Low-Dose Perfusion Computed Tomography: Predicting Prognostic Biomarkers and Molecular Subtypes
Source: Sci Rep. 2019 Nov 28;9:17847. doi: 10.1038/s41598-019-54371-z (PMC6882909; doi:10.1038/s41598-019-54371-z)
Supplement: Supplementary file 1 — Supplementary tables and figure [file 41598_2019_54371_MOESM1_ESM.docx]

**Machine Learning Approaches to Radiogenomics of Breast Cancer using Low-Dose Perfusion Computed Tomography: Predicting Prognostic Biomarkers and Molecular Subtypes**

**Manuscript type:** Original Research

Eun Kyung Park^1^, Kwang-sig Lee^2^, Bo Kyoung Seo^1*^, Kyu Ran Cho^3^, Ok Hee Woo^4^, Gil Soo Son^5^, Hye Yoon Lee^5^, & Young Woo Chang^5^

# Eun Kyung Park and Kwang-sig Lee contributed equally to this article.

^1^Department of Radiology, Korea University Ansan Hospital, Korea University College of Medicine, 123 Jeokgeum-ro, Danwon-gu, Ansan-si, Gyeonggi-do 15355, Republic of Korea

^2^AI Center, Korea University Anam Hospital, Korea University College of Medicine, 73 Inchon-ro, Seongbuk-gu, Seoul 02841, Republic of Korea

^3^Department of Radiology, Korea University Anam Hospital, Korea University College of Medicine, 73, Goryeodae-ro, Seongbuk-gu, Seoul 02841, Republic of Korea

^4^Department of Radiology, Korea University Guro Hospital, Korea University College of Medicine, 148 Gurodong-ro, Guro-gu, Seoul 08308, Republic of Korea

^5^Division of Breast and Endocrine Surgery, Department of Surgery, Korea University Ansan Hospital, Korea University College of Medicine, 123 Jeokgeum-ro, Danwon-gu, Ansan-si, Gyeonggi-do 15355, Republic of Korea

**Correspondence to:** Bo Kyoung Seo, MD, PhD

Department of Radiology, Korea University Ansan Hospital, Korea University College of Medicine, 123 Jeokgeum-ro, Danwon-gu, Ansan city, Gyeonggi-do 15355, Korea.

Tel: +82-31-412-5229, Fax: +82-31-412-5224, E-mail: [seoboky@korea.ac.kr](mailto:seoboky@korea.ac.kr), [seoboky@gmail.com](mailto:seoboky@gmail.com).

**Supplementary Table S1.** Multinomial logistic regression results of the top 5 important CT parameters for predicting prognostic biomarkers and molecular subtypes: Odds ratios for CT parameters on prognostic biomarkers/molecular subtypes

| **CT parameters**^*^ | **Lymph node** | **Tumor grade** | **Tumor size** | **ER** | **PR** | **HER2** | **Ki67** | **Molecular subtype** | | |
| --- | --- | --- | --- | --- | --- | --- | --- | --- | --- | --- |
|  | Positive  vs. negative | High  vs. low | >20  vs. ≤20 | Positive  vs. negative | Positive  vs. negative | Positive  vs. negative | Positive  vs. negative | Luminal B  vs. luminal A | HER2 overexpression  vs. luminal A | Triple negative  vs. luminal A |
| PEI (Hounsfield Units) | 1.0108 | 1.0011 | 1.0292 | 1.0003 | 1.0017 | 1.0184 | 0.9980 | 1.0029 | 1.0026 | 0.9959 |
| TTP (seconds) | 0.9926 | 0.9465 | 0.9779 | 1.0784 | 1.0882 | 0.9412 | 0.9893 | 0.9752 | 0.8792 | 0.9116 |
| BV permeability (mL/100 g) | 0.9885 | 1.0096 | 0.9756 | 0.9955 | 0.9985 | 0.9953 | 1.0392 | 1.0073 | 0.9983 | 1.0116 |
| Perfusion-Function (mL/min per 100 mL) | 1.0068 | 0.9987 | 1.0164 | 0.9949 | 0.9965 | 1.0010 | 1.0084 | 1.0178 | 1.0253 | 1.0136 |
| Perfusion-Function-Whole (mL/min per 100 mL) | 0.9830 | 1.0013 | 0.9558 | 1.0099 | 0.9928 | 1.0103 | 0.9919 | 0.9496 | 0.9764 | 0.9571 |

^*^The meanings of CT parameters are described in Table 1.

*CT* computed tomography, *ER* estrogen receptor, *PR* progesterone receptor, *HER2* human epidermal growth factor receptor 2.

**Supplementary Table S2.** Univariate analysis results of the top 5 important CT parameters for predicting prognostic biomarkers and molecular subtypes: Means and standard deviations for CT parameters on prognostic biomarkers/molecular subtypes

| **CT parameter/lymph node** | **Positive** | | **Negative** | |
| --- | --- | --- | --- | --- |
|  | Mean | SD | Mean | SD |
| PEI | 111.29 | 35.19 | 113.30 | 32.43 |
| TTP | 48.01 | 8.64 | 48.23 | 8.13 |
| Perm BV | 13.85 | 20.74 | 12.55 | 11.61 |
| Perfusion-Function | 33.28 | 30.54 | 29.83 | 32.23 |
| Perfusion-Function-Whole^*^ | 15.47 | 18.67 | 9.62 | 13.82 |
| **CT parameter/tumor grade** | **Low** | | **High** | |
|  | Mean | SD | Mean | SD |
| PEI^*^ | 105.95 | 35.10 | 122.92 | 29.17 |
| TTP^*^ | 49.87 | 8.08 | 45.02 | 8.12 |
| Perm BV^*^ | 10.94 | 11.22 | 17.40 | 24.26 |
| Perfusion-Function^*^ | 26.82 | 30.07 | 40.47 | 31.56 |
| Perfusion-Function-Whole^*^ | 9.70 | 13.29 | 18.65 | 20.88 |
| **CT parameter/tumor size** | **≤20** | | **>20** | |
|  | Mean | SD | Mean | SD |
| PEI^*^ | 104.03 | 31.82 | 120.20 | 34.28 |
| TTP^*^ | 49.22 | 8.21 | 47.00 | 8.50 |
| Perm BV | 12.85 | 22.28 | 13.74 | 10.67 |
| Perfusion-Function^*^ | 28.11 | 30.58 | 35.47 | 31.62 |
| Perfusion-Function-Whole | 13.37 | 18.49 | 12.57 | 15.42 |
| **CT parameter/molecular subtype** | **Luminal A** | | **Luminal B** | |
|  | Mean | SD | Mean | SD |
| PEI^*^ | 102.39 | 37.48 | 113.76 | 31.26 |
| TTP^*^ | 51.14 | 8.09 | 49.11 | 8.05 |
| Perm BV^*^ | 9.70 | 10.60 | 13.58 | 13.44 |
| Perfusion-Function^*^ | 22.57 | 23.85 | 30.14 | 32.73 |
| Perfusion-Function-Whole^*^ | 9.50 | 13.56 | 10.22 | 12.47 |
| **CT parameter/molecular subtype** | **HER2 overexpression** | | **Triple negative** | |
|  | Mean | SD | Mean | SD |
| PEI^*^ | 133.46 | 26.00 | 116.00 | 26.11 |
| TTP^*^ | 41.43 | 6.46 | 44.45 | 6.82 |
| Perm BV^*^ | 19.37 | 8.95 | 16.58 | 32.63 |
| Perfusion-Function^*^ | 53.90 | 33.43 | 39.26 | 32.78 |
| Perfusion-Function-Whole^*^ | 25.90 | 26.44 | 15.74 | 16.60 |

*The p value of the t or F statistic < 0.05

^†^The meanings of CT parameters are described in Table 1.

*CT* computed tomography, *SD* standard deviation*, HER2* human epidermal growth factor receptor 2.

**Supplementary Figure S1.** Areas under receiver-operating-characteristic curves of the top 5 important CT parameters for predicting prognostic biomarkers and molecular subtypes

|  | **Logistic Regression** | **Decision Tree** |
| --- | --- | --- |
| Lymph node | 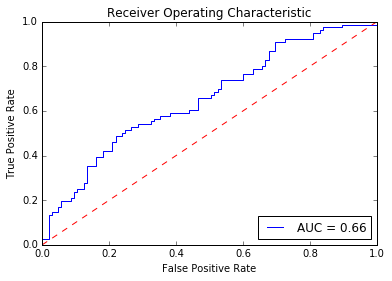 | 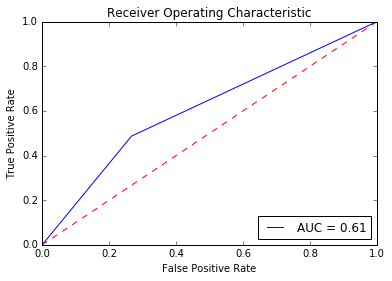 |
| Tumor grade | 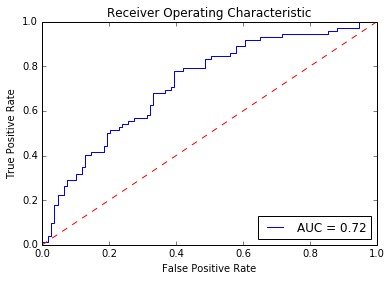 | 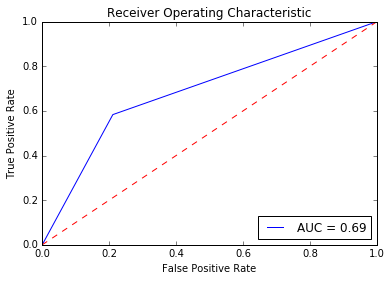 |
| Tumor size | 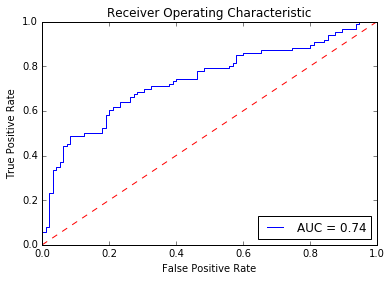 | 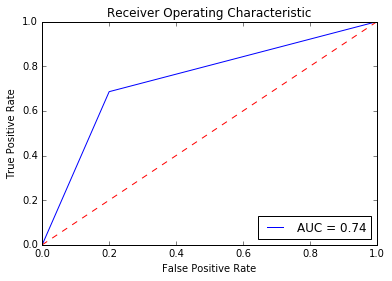 |
| Molecular subtype (Luminal A vs. Luminal B) | 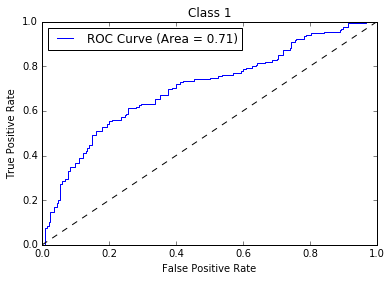 | 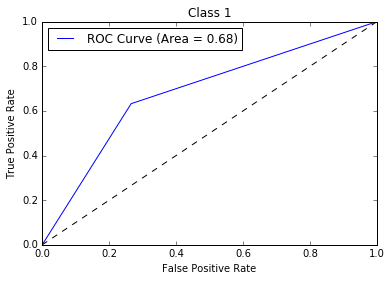 |
| Molecular subtype (Luminal A vs. HER2 verexpression) | 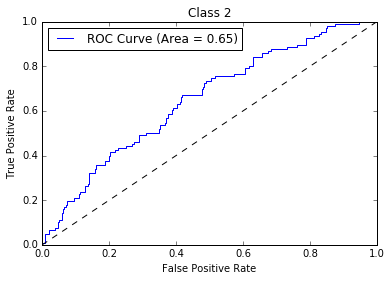 | 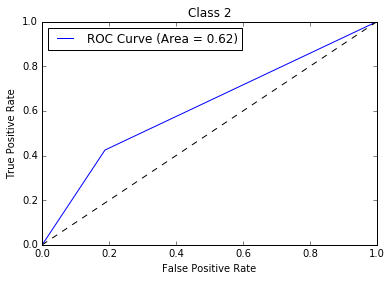 |
| Molecular subtype (Luminal A vs. Triple Negative) | 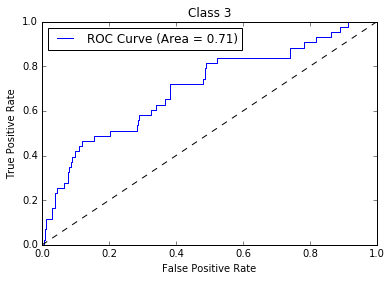 | 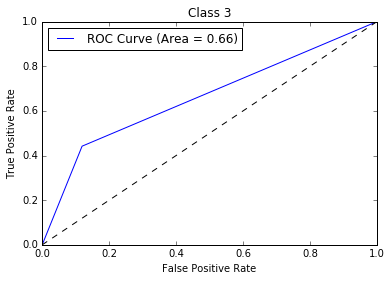 |
|  | **Naïve Bayes** | **Random Forest** |
| Lymph node | 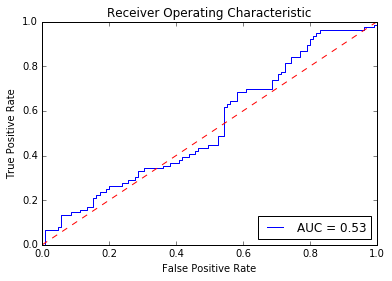 | 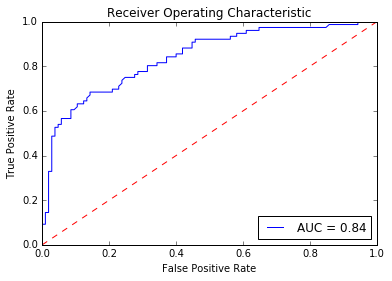 |
| Tumor grade | 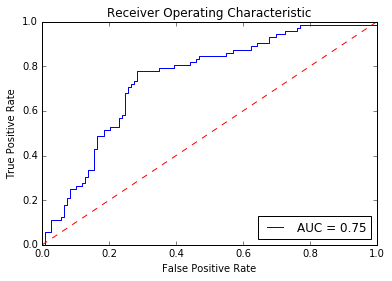 | 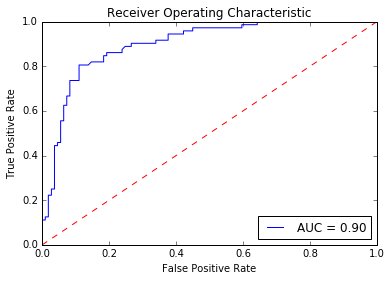 |
| Tumor size | 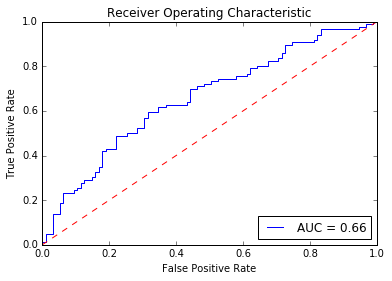 | 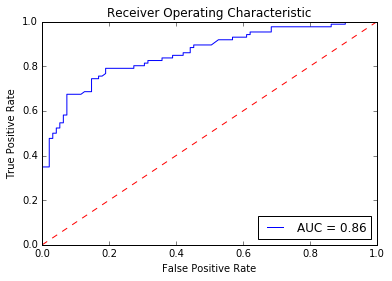 |
| Molecular subtype (Luminal A vs. Luminal B) | 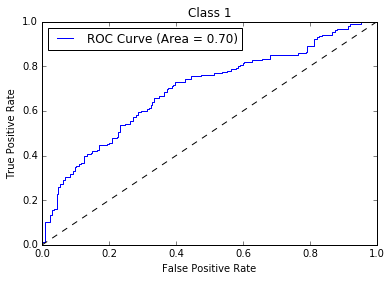 | 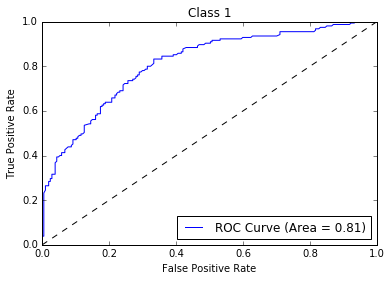 |
| Molecular subtype (Luminal A vs. HER2 Overexpression) | 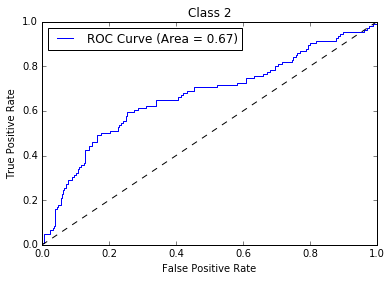 | 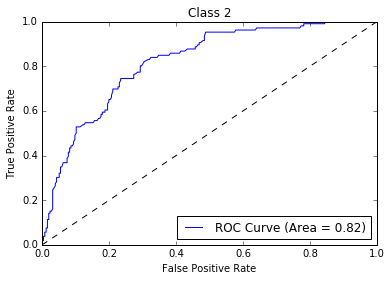 |
| Molecular subtype (Luminal A vs. Triple Negative) | 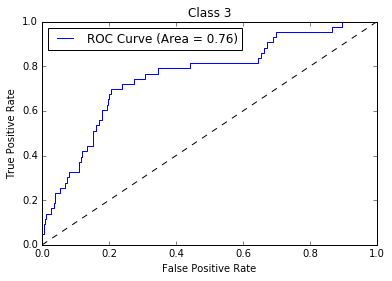 | 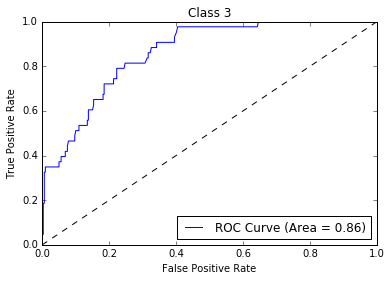 |

|  | **Support Vector Machine** | **Artificial Neural Network** |
| --- | --- | --- |
| Lymph node | 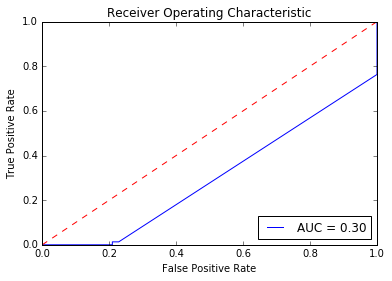 | 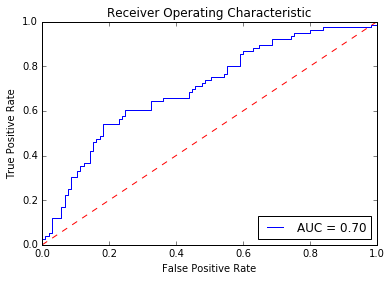 |
| Tumor grade | 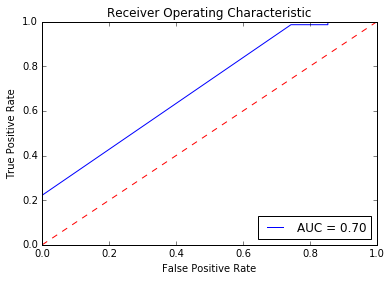 | 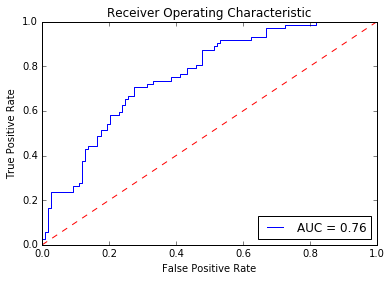 |
| Tumor size | 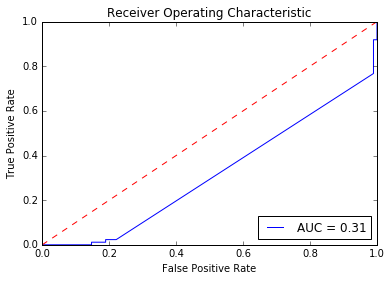 | 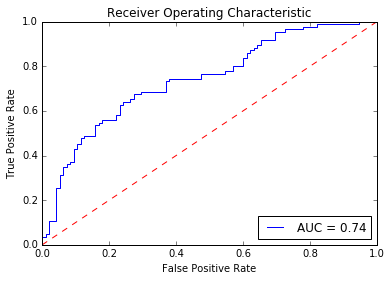 |
| Molecular subtype (Luminal A vs. Luminal B) | 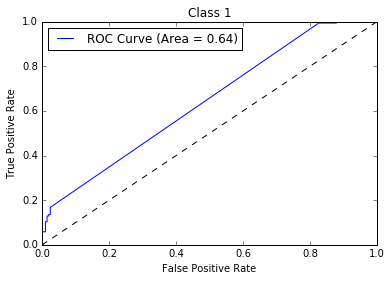 | 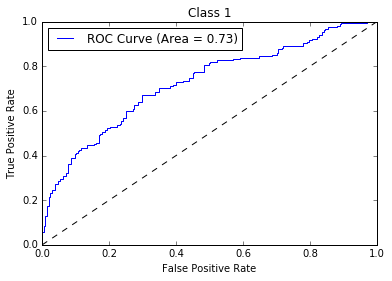 |
| Molecular subtype (Luminal A vs. HER2 Overexpression) | 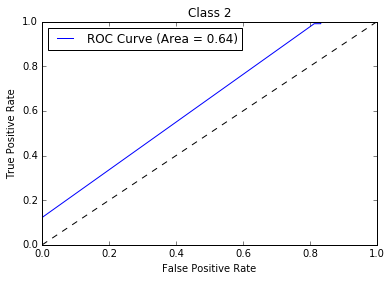 | 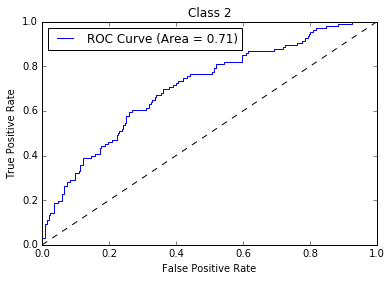 |
| Molecular subtype (Luminal A vs. Triple Negative) | 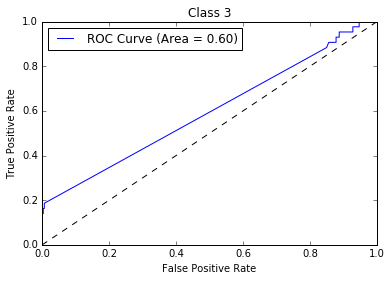 | 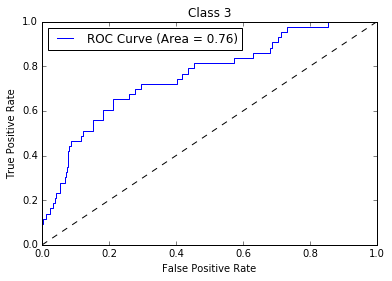 |
